# Supplementary material for: Discovery of an antivirulence compound that targets the Staphylococcus aureus SaeRS two-component system to inhibit toxic shock syndrome toxin-1 production
Source: J Biol Chem. 2024 Jun 7;300(7):107455. doi: 10.1016/j.jbc.2024.107455 (PMC11328871; doi:10.1016/j.jbc.2024.107455)
Supplement: Supporting Information [file mmc1.pdf]

SUPPORTING INFORMATION

Discovery of an antivirulence compound that targets the *Staphylococcus aureus* SaeRS two-component system to inhibit toxic shock syndrome toxin-1 production

Karine Dufresne[1], Dennis A. DiMaggio Jr[2], Carla S. Maduta[1], Shaun R. Brinsmade[2], John K. McCormick[1]

[1]Department of Microbiology and Immunology, University of Western Ontario, London, Ontario, Canada

[2]Department of Biology, Georgetown University, Washington, DC, USA

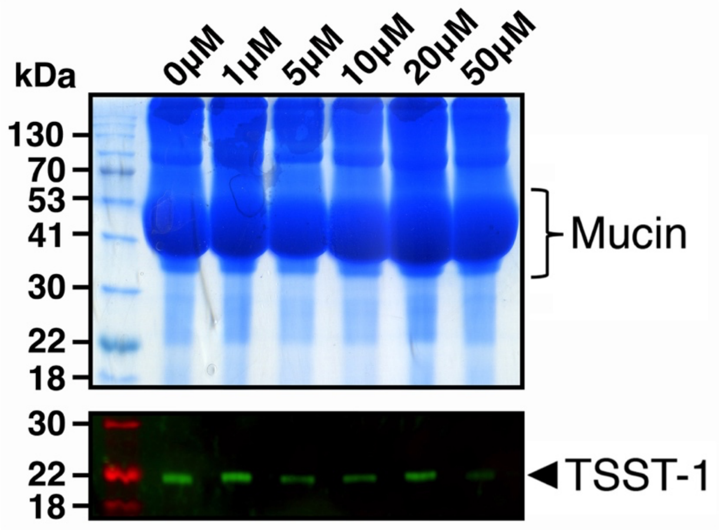

Figure S1. PP-HCl decreases TSST-1 production at 18-hours incubation. TSST-1 production in the supernatants of wild-type *S. aureus* MN8 was evaluated by Western Blot at the same concentrations of PP-HCl as tested during the luciferase assay (Fig 2). Supernatants were harvested after a 18-hours incubation in VDM, concentrated using trichloroacetic acid and normalized to 12 OD_{600} units. Shown are exoprotein profiles (top panels) and Western blot analysis (bottom panels) of TSST-1 for wild-type *S. aureus* MN8.

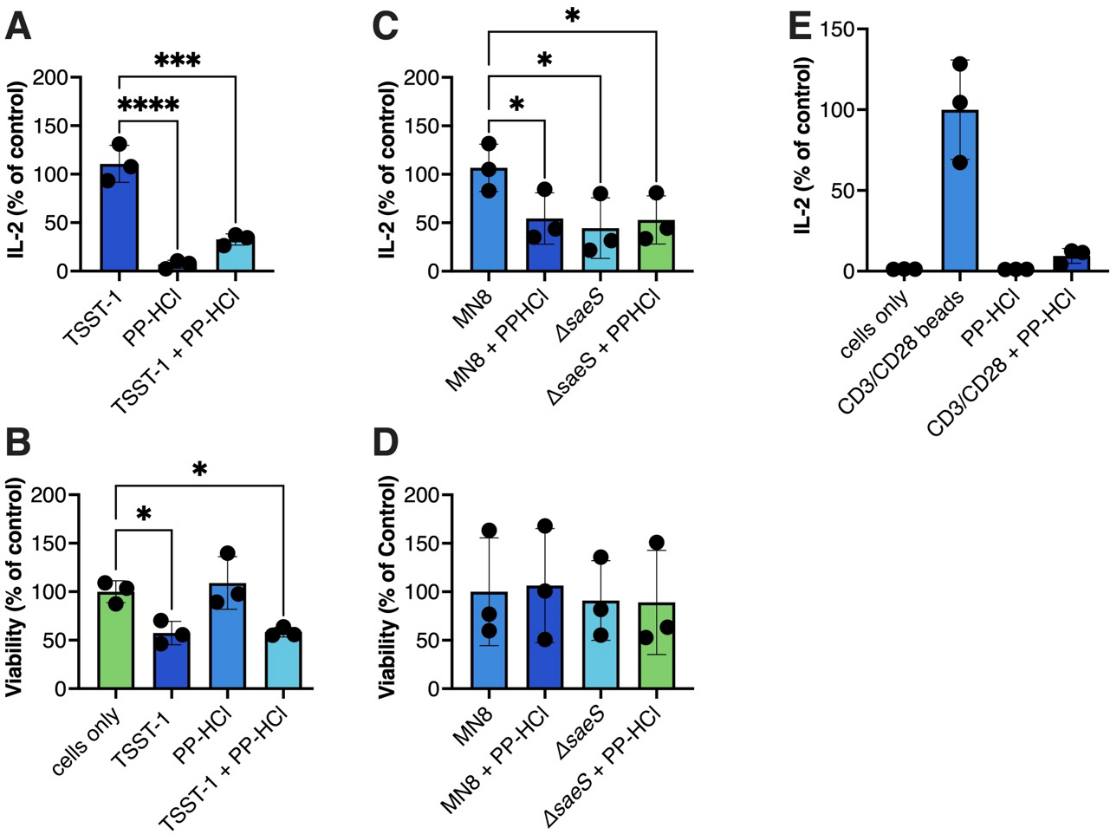

Figure S2. PP-HCl decreases TSST-1-induced IL-2 production from PBMC. (A) Percentage of IL-2 compared to control condition (100 ng/ml TSST-1) was measured for each condition. Ordinary one-way ANOVA was performed (***, $p < 0.001$; ****. $p < 0.0001$). The results are presented as the mean of percentage of IL-2 $\pm$ SD. (B) Percentage of viability compared to the control condition (cells only) was measured for each condition. Ordinary one-way ANOVA was performed (*, $p \leq 0.05$). The results are presented as the mean of percentage of viability $\pm$ SD. (C) Percentage of IL-2 production at a dilution factor of 6250 was compared to the *S. aureus* MN8 supernatant control. Ordinary one-way ANOVA was performed. The results are presented as the mean of percentage of IL-2 $\pm$ SD. (D) Percentage of viability compared to the *S. aureus* MN8 supernatant condition was measured for each condition. Ordinary one-way ANOVA was performed. The results are presented as the mean of percentage of viability $\pm$ SD. (E) Percentage of IL-2 compared to control condition (CD3/CD25 DynaBeads) was measured for each condition. Ordinary one-way ANOVA was performed (***, $p < 0.001$; ****. $p < 0.0001$). The results are presented as the mean of percentage of IL-2 $\pm$ SD.

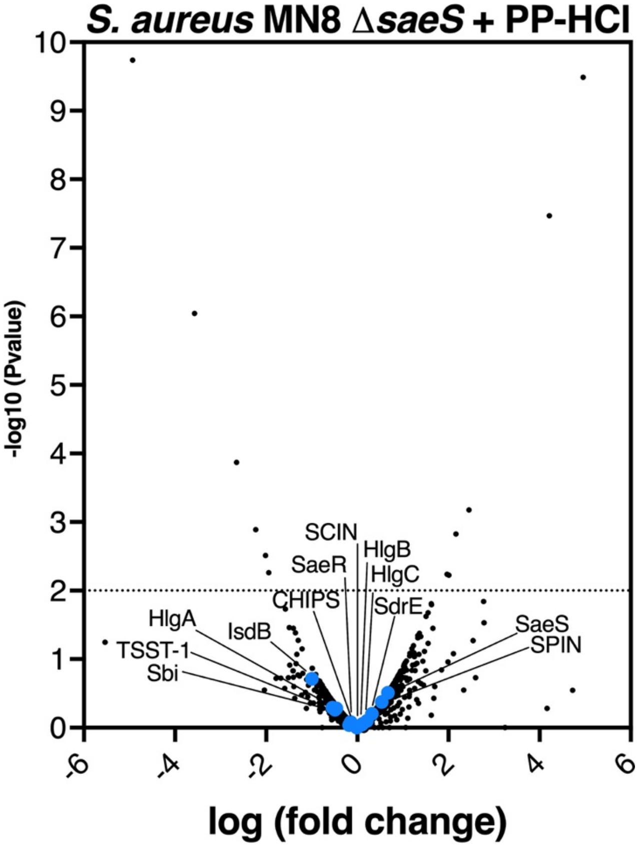

Figure S3. Inactivation of the SaeRS regulon decreases virulence factor expression in *S. aureus* MN8 similar to the addition of PP-HCl. RNAseq comparing *S. aureus* MN8 versus MN8 *ΔsaeS* in 700 μM glucose VDM was analyzed. The main virulence factors associated with SaeRS regulon, also identified in Figure 5, are presented with blue dots.

Table S1. Significant transcriptional comparison analysis of all differentially expressed genes in between *S. aureus* MN8 with or without PP-HCl

Locus tag	Gene name	Description	Log (Fold Change)	P-value	False Discovery Rate
NLA26_RS00140		chemotaxis-inhibiting protein CHIPS	-2,24302619	0,004935101	0,462313203
NLA26_RS00160		hypothetical protein	-3,659580928	1,95E-06	0,000852383
NLA26_RS02710	isdB	heme uptake protein IsdB	-1,769367438	0,012306881	0,896693043
NLA26_RS02845		fibrinogen-binding protein	-1,599693106	0,02215777	1
NLA26_RS02870	scc	complement inhibitor SCIN-C	-2,39882216	0,001344988	0,162433602
NLA26_RS02885	hyl	alpha-hemolysin	-3,828206866	1,70E-07	0,000149017
NLA26_RS03150		hypothetical protein	1,740261214	0,00979148	0,778274301
NLA26_RS04210		phosphate ABC transporter substrate-binding protein PstS	1,372391329	0,047233419	1
NLA26_RS04785		tail protein	2,766440303	0,007289357	0,616773681
NLA26_RS06340		AAA family ATPase	-3,847933922	1,20E-06	0,000629896
NLA26_RS06345		DDE-type integrase/transposase/recombinase	3,57819408	2,67E-06	0,000876259
NLA26_RS06515		IS3 family transposase	-2,859483473	0,001347849	0,162433602
NLA26_RS07110		DM13 domain-containing protein	-2,642615584	0,00014624	0,027399133
NLA26_RS07115		DoxX family protein	-2,439377481	0,000417208	0,068396028
NLA26_RS07120	saeR	response regulator transcription factor SaeR	-1,521562381	0,022923182	1
NLA26_RS07285		transposase	-2,806802315	0,029588432	1
NLA26_RS07820	adhP	alcohol dehydrogenase AdhP	1,66546417	0,013414396	0,925946341
NLA26_RS07845		aldo/keto reductase	3,001823827	2,04E-05	0,00486424
NLA26_RS07985		MFS transporter	1,393914093	0,036328038	1
NLA26_RS08035	sdrE	MSCRAMM family adhesin SdrE	2,616281758	0,000162171	0,028358307
NLA26_RS08665		ABC transporter permease	2,173488812	0,029323086	1
NLA26_RS08710		DUF2294 domain-containing protein	1,370404433	0,04093036	1
NLA26_RS08715		YbcC family protein	2,347450254	0,000631615	0,097454556
NLA26_RS08720		NADH dehydrogenase subunit 5	3,108270586	1,23E-05	0,003218903
NLA26_RS08805	spn	myeloperoxidase inhibitor SPIN	-1,942376963	0,004116629	0,415304568
NLA26_RS08830		hypothetical protein	11,97357639	4,96E-16	1,30E-12
NLA26_RS08845		superantigen-like protein SSL7	-1,811789693	0,021411536	1
NLA26_RS09055		terminase small subunit	-1,627938062	0,038020906	1
NLA26_RS09330		PTS ascorbate transporter subunit IIC	1,986597389	0,003435188	0,360419915
NLA26_RS09460		formate/nitrite transporter family protein	1,804605025	0,007836755	0,642368993
NLA26_RS09780		PTS transporter subunit EIIC	1,506045462	0,026572905	1
NLA26_RS09785		L-lactate dehydrogenase	1,69511943	0,011536168	0,864553393
NLA26_RS09800		hypothetical protein	1,537702319	0,022714696	1
NLA26_RS09805		DUF488 domain-containing protein	1,911986743	0,005807458	0,525274541
NLA26_RS09880	pflA	pyruvate formate-lyase-activating protein	1,510539169	0,024662812	1
NLA26_RS09885	pflB	formate C-acetyltransferase	2,261360621	0,000949328	0,138338252
NLA26_RS09910		isoprenylcysteine carboxyl methyltransferase family protein	-3,504918267	2,69E-05	0,005870903
NLA26_RS10110		ABC transporter ATP-binding protein	3,199782966	0,004550577	0,44208016
NLA26_RS10225	adhE	bifunctional acetaldehyde-CoA/alcohol dehydrogenase	2,117597635	0,002181478	0,238417346
NLA26_RS11185		glutathione peroxidase	-1,407029256	0,036103814	1
NLA26_RS11200		NAD(P)-binding protein	-1,476126369	0,027856441	1
NLA26_RS11210		CitMHS family transporter	-2,842911684	4,94E-05	0,009961835

NLA26_RS11290		hypothetical protein	1,318450015	0,049944002	1
NLA26_RS11645		hypothetical protein	1,587481609	0,022400495	1
NLA26_RS11695		D-lactate dehydrogenase	1,558244497	0,019759696	1
NLA26_RS11720		ring-cleaving dioxygenase	-1,719592911	0,04127558	1
NLA26_RS11790		MerR family transcriptional regulator	2,207672109	0,001235584	0,162433602
NLA26_RS11870		hypothetical protein	1,458076504	0,028932455	1
NLA26_RS11940	cntL	D-histidine (S)-2-aminobutanoyltransferase CntL	-1,954899084	0,023294179	1
NLA26_RS12135		ABC transporter ATP-binding protein/permease	1,738193519	0,010951952	0,84491089
NLA26_RS12180	hlgB	bi-component gamma-hemolysin HlgAB/HlgCB subunit B	-1,341484857	0,048399452	1
NLA26_RS12190	hlgA	bi-component gamma-hemolysin HlgAB subunit A	-1,378755834	0,045414938	1
NLA26_RS12195	sbi	immunoglobulin-binding protein Sbi	-3,226248139	1,06E-05	0,0030989
NLA26_RS12210		2,3-diphosphoglycerate-dependent phosphoglycerate mutase	2,134177701	0,001721042	0,196273659
NLA26_RS12300	nirB	nitrite reductase large subunit NirB	1,498957138	0,025448806	1
NLA26_RS12335	nreA	nitrate respiration regulation accessory nitrate sensor NreA	1,451777396	0,029655697	1
NLA26_RS12345	nreC	nitrate respiration regulation response regulator NreC	-1,508402286	0,024125403	1
NLA26_RS12385		IS1182-like element ISSau3 family transposase	-1,675320076	0,014161013	0,952418919
NLA26_RS12475		L-lactate permease	1,603332658	0,016609813	1
NLA26_RS12500		DUF3021 domain-containing protein	3,448503028	2,58E-06	0,000876259
NLA26_RS12505		LytTR family DNA-binding domain-containing protein	4,222707423	2,39E-08	3,14E-05
NLA26_RS13390		lactose-specific PTS transporter subunit EIIC	-1,661919394	0,01675983	1
NLA26_RS13590	fmtB	LPXTG-anchored DUF1542 repeat protein FmtB	1,640571816	0,015311673	1
NLA26_RS13765		UDP-N-acetylglucosamine 1-carboxyvinyltransferase	-2,18598313	0,001362386	0,162433602
NLA26_RS14375	tst	toxic shock syndrome toxin TSST-1	-3,585464999	8,20E-07	0,00053791
NLA26_RS14390		TDT family transporter	1,428978692	0,033704317	1

Table S2. Significant transcriptional comparison analysis of all differentially expressed genes in between *S. aureus* MN8 and MN8Δ*saeS*

Locus tag	Gene name	Description	log(Fold Change)	P-Value	False Discovery Rate
NLA26_RS00160		hypothetical protein	-5.37677009051546	2,64E-09	1.39002024595471e-06
NLA26_RS00165	eap	extracellular adherence protein Eap/Map	-1.64472645426634	0,01480866	0.304618760750577
NLA26_RS00330	purB	adenylosuccinate lyase	-1.34109712715389	0,043888166	0.520529466414903
NLA26_RS00415	ftnA	H-type ferritin FtnA	1.46455361123511	0,028075047	0.420009079569682
NLA26_RS00470		YihY/virulence factor BrkB family protein	1.96070525418312	0,003774294	0.130759433584258
NLA26_RS00480		low molecular weight phosphotyrosine protein phosphatase	1.31714561235869	0,048893675	0.554577244337202
NLA26_RS00520		hypothetical protein	-2.09546974152156	0,00284159	0.110028020928462
NLA26_RS01115		glucosamine-6-phosphate isomerase	1.67292047214202	0,012652309	0.278484695787963
NLA26_RS01120		SAS053 family protein	2.02091202043348	0,002914119	0.111201083586865
NLA26_RS01125		hypothetical protein	1.64450400406338	0,015991033	0.309839051146738
NLA26_RS01330		DUF4041 domain-containing protein	-1.57281111067916	0,027837232	0.418831032091609
NLA26_RS01335		membrane protein	-2.96342588002338	0,002484979	0.0991356182297862
NLA26_RS01340		serine protease	1.68850434347613	0,04221484	0.512219694073386
NLA26_RS01350		serine protease	1.59594839915989	0,022651597	0.372760350911784
NLA26_RS01390		hypothetical protein	-1.37593140464848	0,041407976	0.508931688818941
NLA26_RS01600		FAD/NAD(P)-binding domain-containing protein	1.89883487153256	0,005005887	0.146450012307579
NLA26_RS01715		BlaZ family penicillin-hydrolyzing class A beta-lactamase PC1	-2.55685951486929	0,000924321	0.0517816378106167
NLA26_RS02185		lipoate--protein ligase	-1.62956854709113	0,015915429	0.309839051146738
NLA26_RS02240		DoxX family protein	-1.6442792992403	0,016574835	0.313967911154599
NLA26_RS02390	purE	5-(carboxyamino)imidazole ribonucleotide mutase	-3.53490791380687	1,46E-06	0.000274116762880999
NLA26_RS02395	purK	5-(carboxyamino)imidazole ribonucleotide synthase	-3.25424577503941	5,12E-06	0.000842997512996677
NLA26_RS02400		phosphoribosylaminoimidazolesuccinocarboxamide synthase	-2.64003237330146	0,000151926	0.0121218136818305
NLA26_RS02405	purS	phosphoribosylformylglycinamidine synthase subunit PurS	-2.17765978165622	0,001510895	0.0765035902390358
NLA26_RS02410	purQ	phosphoribosylformylglycinamidine synthase I	-1.88949613287097	0,00520944	0.148114643542223
NLA26_RS02460	graF	glycopeptide resistance-associated protein GraF	1.76438313251934	0,018375679	0.338343801178553
NLA26_RS02475		hypothetical protein	1.69400102787375	0,011698604	0.267847171283806
NLA26_RS02480		phosphocarrier protein HPr	1.34592378984723	0,042982749	0.516774326015132
NLA26_RS02500		cytochrome d ubiquinol oxidase subunit II	1.96388439733587	0,004142066	0.13308265321798
NLA26_RS02600		YktB family protein	-1.92633345871556	0,004637587	0.13875871091136
NLA26_RS02645		heme A synthase	1.36754891411209	0,040043338	0.500177543140077
NLA26_RS02710	isdB	heme uptake protein IsdB	-1.74124430327641	0,014223736	0.299608779099668
NLA26_RS02720	isdC	heme uptake protein IsdC	-2.16079854023557	0,004126092	0.13308265321798
NLA26_RS02725	isdD	iron-regulated surface determinant protein IsdD	-1.54732813805994	0,040272556	0.500177543140077
NLA26_RS02730	isdE	heme ABC transporter substrate-binding protein IsdE	-1.81006008703195	0,01299625	0.282802687126705
NLA26_RS02845		fibrinogen-binding protein	-4.23020520610729	8,00E-07	0.000162002379875929
NLA26_RS02865	efb	complement convertase inhibitor Efb	-3.92545068110679	0,002294842	0.0959098300588847
NLA26_RS02870	scc	complement inhibitor SCIN-C	-3.50588048556493	4,31E-05	0.00472713241522785
NLA26_RS02885	hyl	alpha-hemolysin	-6.81567180853663	9,70E-16	2.55474945112447e-12
NLA26_RS02890		hypothetical protein	-2.38703980738747	0,012065199	0.269217530498303
NLA26_RS02930		YfcC family protein	1.59689147876159	0,01746787	0.326190793903062
NLA26_RS03075	pyrR	bifunctional pyr operon transcriptional regulator/uracil phosphoribosyltransferase PyrR	-2.86353899676997	5,74E-05	0.00563609486990349
NLA26_RS03080		NCS2 family nucleobase:cation symporter	-1.94999982276432	0,004617428	0.13875871091136

NLA26_RS03150		hypothetical protein	-1.39684485782387	0,037875107	0.496831513021077
NLA26_RS03660		hypothetical protein	1.45242151522687	0,039822284	0.500177543140077
NLA26_RS03730		hypothetical protein	-1.85085447617534	0,013427989	0.287446304362568
NLA26_RS03735		hypothetical protein	-2.55123224179508	0,000742257	0.0437076636491352
NLA26_RS03800		hypothetical protein	-2.44929211208228	0,039696281	0.500177543140077
NLA26_RS03940	guaC	GMP reductase	-1.46475592953867	0,029234559	0.424321446252027
NLA26_RS03950		CAP domain-containing protein	-1.72426981374956	0,010472271	0.244013191075248
NLA26_RS04075		IS1182-like element ISSau3 family transposase	-3.17285088466373	0,019786881	0.346231055404228
NLA26_RS04080		4-oxalocrotonate tautomerase	-1.75497119405364	0,021988611	0.371128278669463
NLA26_RS04100		anthranilate synthase component I	-1.4450458269613	0,04551632	0.531864534783546
NLA26_RS04180		hypothetical protein	-1.49282316746461	0,030297164	0.424321446252027
NLA26_RS04185	pepF	oligoendopeptidase F	-1.45462532012685	0,029238889	0.424321446252027
NLA26_RS04195	pstB	phosphate ABC transporter ATP-binding protein PstB	1.62329135045013	0,022297572	0.371579161326147
NLA26_RS04205	pstC	phosphate ABC transporter permease subunit PstC	1.54769823976672	0,03975818	0.500177543140077
NLA26_RS04230		aspartate-semialdehyde dehydrogenase	1.65358209924865	0,01600384	0.309839051146738
NLA26_RS04265	msaC	sarA expression modulator MsaC	-2.21095471708566	0,002242598	0.0959098300588847
NLA26_RS04295	brnQ	branched-chain amino acid transport system II carrier protein	-1.52300975855829	0,023611975	0.381413073931117
NLA26_RS04335	arlR	response regulator transcription factor ArlR	2.93545166735638	3,05E-05	0.00365437963903845
NLA26_RS04405		thymidylate synthase	1.48401600830932	0,026290957	0.40960999650667
NLA26_RS04435		ribonuclease HI family protein	-2.28461842956687	0,012692048	0.278484695787963
NLA26_RS05010		hypothetical protein	-2.41987467372493	0,003937636	0.133069958568047
NLA26_RS05255		aminopeptidase P family protein	-1.57476851219884	0,018903389	0.345643212617779
NLA26_RS05330		competence type IV pilus ATPase ComGA	1.66093141456811	0,024434295	0.392289618553025
NLA26_RS05350		YqgQ family protein	4.12195095786939	3,36E-08	1.26217534693628e-05
NLA26_RS05410		tRNA (adenine(22)-N(1))-methyltransferase TrmK	1.64453118081587	0,015195339	0.305414716778722
NLA26_RS05715		LLM class flavin-dependent oxidoreductase	-1.35021089813828	0,045082175	0.529916810893787
NLA26_RS05985		amino acid permease	2.26030355788642	0,000960076	0.0526641848757118
NLA26_RS06145	tpx	thiol peroxidase	-1.31942275014202	0,047308535	0.543944862668992
NLA26_RS06240	harA	haptoglobin-binding heme uptake protein HarA	1.46112230331633	0,033114959	0.45412337508229
NLA26_RS06340		AAA family ATPase	-4.69493748108572	4,35E-08	1.43081759880947e-05
NLA26_RS06350		recombinase family protein	-2.78747751216465	0,029721761	0.424321446252027
NLA26_RS06365		BlaZ family penicillin-hydrolyzing class A beta-lactamase PC1	-2.88847374406505	8,67E-05	0.00761207308549562
NLA26_RS06380		Na+/H+ antiporter family protein	1.48987803575207	0,027196804	0.413925928232178
NLA26_RS06385		M17 family metallopeptidase	-1.52015257974271	0,023086208	0.375303540824621
NLA26_RS06445		hypothetical protein	-1.99902580340462	0,018080665	0.335256276067419
NLA26_RS06515		IS3 family transposase	-2.8568846225394	0,004025529	0.13308265321798
NLA26_RS06595		thioredoxin family protein	-1.54153034632549	0,021469073	0.367065377049659
NLA26_RS06610		organic hydroperoxide resistance protein	1.45019934453155	0,030194819	0.424321446252027
NLA26_RS06670		thermonuclease family protein	-3.03424752201478	2,57E-05	0.00355601454130274
NLA26_RS06690	clfA	MSCRAMM family adhesin clumping factor ClfA	1.75444135044125	0,009066869	0.224188662838043
NLA26_RS06700		hypothetical protein	1.41422091907711	0,040168919	0.500177543140077
NLA26_RS06715		stage II sporulation protein M	-1.87514894273527	0,011987912	0.269217530498303
NLA26_RS06780		DUF4887 domain-containing protein	1.86973908431204	0,009110591	0.224188662838043
NLA26_RS06785		TIGR01777 family oxidoreductase	2.15307546641194	0,001588479	0.07891446245122
NLA26_RS07020		diacylglycerol kinase family lipid kinase	1.88446692158012	0,0052878	0.148114643542223
NLA26_RS07035		ABC transporter permease/substrate-binding protein	1.38289491386829	0,03792751	0.496831513021077
NLA26_RS07040		ABC transporter ATP-binding protein	1.99908142028848	0,003316822	0.121294319879618

NLA26_RS07110		DM13 domain-containing protein	-4.97662306752756	1,73E-10	1.13651160963298e-07
NLA26_RS07115		DoxX family protein	-4.18117069018232	2,73E-08	1.1996166027104e-05
NLA26_RS07120	saeR	response regulator transcription factor SaeR	-2.07776197239711	0,002283731	0.0959098300588847
NLA26_RS07125	saeS	two-component system sensor histidine kinase SaeS	-2.20861511297373	0,00122708	0.0659367629924807
NLA26_RS07200		DNA photolyase family protein	1.75357079366749	0,00920847	0.224499099532798
NLA26_RS07245		hypothetical protein	1.8438978744328	0,006306143	0.172959103564771
NLA26_RS07250		YaiI/YqxD family protein	1.88550653597938	0,00527479	0.148114643542223
NLA26_RS07255		TIGR00730 family Rossman fold protein	1.90299891820889	0,004864477	0.143911995818681
NLA26_RS07265		hypothetical protein	1.4024888613214	0,036248098	0.485655197069209
NLA26_RS07335		LysR family transcriptional regulator	1.44873034794227	0,030092332	0.424321446252027
NLA26_RS07445		hypothetical protein	-2.29276119240133	0,0064056	0.173875706384279
NLA26_RS07670		metal ABC transporter ATP-binding protein	-1.42078518961471	0,03292485	0.453880262731436
NLA26_RS07820	adhP	alcohol dehydrogenase AdhP	3.42515093198433	1,92E-06	0.000336862957294798
NLA26_RS07895		phosphomevalonate kinase	1.48949888462704	0,025749191	0.405973765170822
NLA26_RS07900	mvaD	diphosphomevalonate decarboxylase	2.00899604101226	0,003075112	0.114039014617043
NLA26_RS07905	mvk	mevalonate kinase	1.39563652766579	0,036336526	0.485655197069209
NLA26_RS07920		heme-dependent peroxidase	-1.38534323935443	0,037532936	0.496604118208844
NLA26_RS07935		APC family permease	1.44212772027937	0,030495927	0.424845369755507
NLA26_RS08040		MSCRAMM family adhesin SdrD	-2.01647336457745	0,002979711	0.112079702497114
NLA26_RS08045	sdrC	MSCRAMM family adhesin SdrC	-1.6806660296492	0,01327251	0.286446868788349
NLA26_RS08095	hchA	protein deglycase HchA	1.38832859070628	0,038901496	0.500177543140077
NLA26_RS08450		hypothetical protein	4.91649249833108	1,60E-07	4.21889658930087e-05
NLA26_RS08460		RidA family protein	1.6278773777076	0,015095551	0.305414716778722
NLA26_RS08470	ispE	4-(cytidine 5'-diphospho)-2-C-methyl-D-erythritol kinase	1.48982089576414	0,025738083	0.405973765170822
NLA26_RS08585		hypothetical protein	3.67726907831664	0,019786881	0.346231055404228
NLA26_RS08620	gltB	glutamate synthase large subunit	1.48579917744966	0,027499672	0.41613008801993
NLA26_RS08665		ABC transporter permease	2.52355171456433	0,014413706	0.301200689280941
NLA26_RS08670		methionine ABC transporter ATP-binding protein	1.80429011643634	0,019719872	0.346231055404228
NLA26_RS08725		phenol-soluble modulin PSM-alpha-1	-2.16878011198727	0,003571198	0.128271749284151
NLA26_RS08805	spn	myeloperoxidase inhibitor SPIN	-3.76990874964928	2,54E-07	6.07125249479207e-05
NLA26_RS08840		superantigen-like protein SSL9	-2.30082674100581	0,045651874	0.531864534783546
NLA26_RS08845		superantigen-like protein SSL7	-1.74372514878465	0,028556984	0.42401216515305
NLA26_RS08925		purine permease	-1.34903981506131	0,042778457	0.516677422692211
NLA26_RS08930	xpt	xanthine phosphoribosyltransferase	-1.49024049799704	0,026267224	0.40960999650667
NLA26_RS08960		NADPH-dependent oxidoreductase	-1.74843805190154	0,00938111	0.224549659034828
NLA26_RS08965	ahpC	alkyl hydroperoxide reductase subunit C	-1.53937947155835	0,021208115	0.367065377049659
NLA26_RS08975		hypothetical protein	-1.41191033414795	0,034589363	0.471884935799889
NLA26_RS09010		PepSY domain-containing protein	1.32293296445318	0,04693975	0.542071757389236
NLA26_RS09140		type II toxin-antitoxin system PemK/MazF family toxin	-1.65887429991339	0,01478577	0.304618760750577
NLA26_RS09190		PLP-dependent aspartate aminotransferase family protein	1.46828612970125	0,041057855	0.507536773944345
NLA26_RS09440		ABC transporter ATP-binding protein	1.56057656093593	0,01973536	0.346231055404228
NLA26_RS09445		FtsX-like permease family protein	2.06782336424611	0,00235734	0.096982422123195
NLA26_RS09610		hypothetical protein	-2.81017095542432	0,000324622	0.0213004164483694
NLA26_RS09620		LacI family transcriptional regulator	1.63319601547492	0,015095859	0.305414716778722
NLA26_RS09645		class I SAM-dependent methyltransferase	-1.83400982172708	0,011134052	0.257157530198967
NLA26_RS09830		acyl--CoA ligase	-1.3542157804769	0,041750568	0.508931688818941
NLA26_RS09850		hypothetical protein	-1.66432451566021	0,016270349	0.312699482488758

NLA26_RS09880	pflA	pyruvate formate-lyase-activating protein	2.61594933380526	0,000172018	0.0133212922042356
NLA26_RS09885	pflB	formate C-acetyltransferase	2.14841634500753	0,001618952	0.0789388776264058
NLA26_RS09910		isoprenylcysteine carboxyl methyltransferase family protein	-3.7619590289485	2,95E-05	0.00365437963903845
NLA26_RS10095		acyl-CoA/acyl-ACP dehydrogenase	1.55873814215273	0,027096883	0.413925928232178
NLA26_RS10105		ABC transporter substrate-binding protein	1.8528936290462	0,049075768	0.554577244337202
NLA26_RS10110		ABC transporter ATP-binding protein	2.89424682270709	0,039696281	0.500177543140077
NLA26_RS10205	cap8D	type 8 capsular polysaccharide synthesis protein Cap8D	1.45188550969826	0,029504903	0.424321446252027
NLA26_RS10210	cap8C	type 8 capsular polysaccharide synthesis protein Cap8C	1.96399175450398	0,003733384	0.130759433584258
NLA26_RS10215	cap8B	type 8 capsular polysaccharide synthesis protein Cap8B	1.94076481258988	0,004144617	0.13308265321798
NLA26_RS10220	capA	capsular polysaccharide type 5/8 biosynthesis protein CapA	1.60004091873066	0,017009782	0.319905398568957
NLA26_RS10225	adhE	bifunctional acetaldehyde-CoA/alcohol dehydrogenase	2.45550282305457	0,000439221	0.0275349803902896
NLA26_RS10305		superoxide dismutase	-1.49416805232694	0,041742789	0.508931688818941
NLA26_RS10425		DUF1648 domain-containing protein	1.42823768833984	0,032784865	0.453880262731436
NLA26_RS10620		adenylosuccinate synthase	-2.57191520139	0,00020506	0.0145924822895155
NLA26_RS10665	hutH	histidine ammonia-lyase	-1.8188978947649	0,007016456	0.188142878522771
NLA26_RS10750		cold-shock protein	1.68738818133625	0,011965462	0.269217530498303
NLA26_RS10795		arylamine N-acetyltransferase	-1.38167697245347	0,038197498	0.497891150724751
NLA26_RS10805		DNA-binding protein	1.46564971745449	0,028664704	0.42401216515305
NLA26_RS10820	cna	collagen adhesin Cna	5.37363835603948	9,92E-12	1.30608580489671e-08
NLA26_RS10825	bstA	bacillithiol transferase BstA	1.78034948939466	0,008828353	0.221381457572698
NLA26_RS10840		SMP-30/gluconolactonase/LRE family protein	-1.82395359154035	0,007575413	0.197485770142584
NLA26_RS10975		flavin reductase family protein	1.41813812727049	0,03607992	0.485655197069209
NLA26_RS10980		hypothetical protein	2.65329910573329	0,000140869	0.0115908691680912
NLA26_RS11025	secA2	accessory Sec system translocase SecA2	1.37510888170354	0,039019255	0.500177543140077
NLA26_RS11030	gtfA	accessory Sec system glycosyltransferase GtfA	1.53924303671329	0,021395584	0.367065377049659
NLA26_RS11100	arcA	arginine deiminase	1.57622707311474	0,022469006	0.37208108866958
NLA26_RS11105	argF	ornithine carbamoyltransferase	2.17319128069171	0,002068889	0.0939204077412553
NLA26_RS11185		glutathione peroxidase	-1.57691447812031	0,019692079	0.346231055404228
NLA26_RS11210		CitMHS family transporter	-3.22974718791638	5,95E-06	0.000921495204438831
NLA26_RS11215	nrdD	anaerobic ribonucleoside-triphosphate reductase	1.45409481588586	0,029384281	0.424321446252027
NLA26_RS11220	nrdG	anaerobic ribonucleoside-triphosphate reductase activating protein	2.09102942275498	0,002236565	0.0959098300588847
NLA26_RS11265		antibiotic biosynthesis monooxygenase	1.96997228440269	0,003942065	0.133069958568047
NLA26_RS11290		hypothetical protein	2.80533749828127	6,59E-05	0.00598148983015147
NLA26_RS11295		aspartate aminotransferase family protein	2.91253021409322	4,84E-05	0.00509358626729982
NLA26_RS11300		amino acid permease	1.98160508354711	0,003605055	0.128271749284151
NLA26_RS11370		fructosamine kinase family protein	2.59185295964013	0,000203429	0.0145924822895155
NLA26_RS11435		VOC family protein	1.36939983838234	0,048425982	0.553733585865085
NLA26_RS11460		PTS transporter subunit IIC	1.9962990370779	0,004410223	0.135715108065041
NLA26_RS11475		CHAP domain-containing protein	2.05691944555524	0,002584104	0.10155143745848
NLA26_RS11480	crtO	glycosyl-4,4'-diaponeurosporenoate acyltransferase	2.59847938544829	0,000190853	0.0143575965228545
NLA26_RS11485		NAD(P)/FAD-dependent oxidoreductase	2.80288488982067	6,04E-05	0.005676666356139
NLA26_RS11560	feoB	ferrous iron transport protein B	-2.247245533595	0,001688108	0.0808143176927569
NLA26_RS11570	clpL	ATP-dependent Clp protease ATP-binding subunit ClpL	1.78821903457925	0,008001955	0.202587958226599
NLA26_RS11610		LrgB family protein	1.80133406928721	0,007489906	0.197209225618275
NLA26_RS11795		GntR family transcriptional regulator	-1.6570018652946	0,026991701	0.413925928232178
NLA26_RS11875		hypothetical protein	3.99566138140707	5,78E-05	0.00563609486990349
NLA26_RS11890		DUF1433 domain-containing protein	2.07674101659351	0,002440859	0.0988735548290392

NLA26_RS11925		SDR family oxidoreductase	2.23970005676555	0,001300788	0.0684995188923924
NLA26_RS11930		MBL fold metallo-hydrolase	1.92970542044556	0,004408047	0.135715108065041
NLA26_RS12000		ATP-binding cassette domain-containing protein	1.61213864659547	0,016546894	0.313967911154599
NLA26_RS12125	gtxA	flippase GtxA	1.8393255175108	0,007921018	0.202485825454381
NLA26_RS12130		type I toxin-antitoxin system Fst family toxin	1.88160577067857	0,007776953	0.200752130031391
NLA26_RS12180	hlgB	bi-component gamma-hemolysin HlgAB/HlgCB subunit B	-1.60735780457233	0,019593487	0.346231055404228
NLA26_RS12185	hlgC	bi-component gamma-hemolysin HlgCB subunit C	-1.83070115357261	0,009539803	0.226290994234177
NLA26_RS12195	sbi	immunoglobulin-binding protein Sbi	-3.25059523905307	1,11E-05	0.00162951414979976
NLA26_RS12220	mdeA	multidrug efflux MFS transporter MdeA	3.99663696284463	6,00E-08	1.75442606722497e-05
NLA26_RS12315		nitrate reductase subunit alpha	2.11219141424649	0,002016212	0.0931348559386545
NLA26_RS12360		NarK/NasA family nitrate transporter	1.35295492352066	0,043718771	0.520529466414903
NLA26_RS12385		IS1182-like element ISSau3 family transposase	-2.5690637180014	0,000331681	0.0213004164483694
NLA26_RS12465		hypothetical protein	1.40350267682029	0,035102548	0.476417575108347
NLA26_RS12660	hutG	formimidoylglutamase	1.4804749911867	0,026611976	0.41217254038676
NLA26_RS12665	fosB	FosB/FosD family fosfomycin resistance bacillithiol transferase	-1.48534371607879	0,04635875	0.537720659644212
NLA26_RS12675	hutU	urocanate hydratase	-2.0777764062212	0,002248533	0.0959098300588847
NLA26_RS12680	hutI	imidazolonepropionase	-1.85462113598284	0,005977377	0.165667730338435
NLA26_RS12785	fdhF	formate dehydrogenase subunit alpha	1.3873974771277	0,037074245	0.493012562872227
NLA26_RS12890		urease subunit beta	-2.07201738083077	0,007074115	0.188142878522771
NLA26_RS12895		urease subunit gamma	-3.33123099096775	0,00138806	0.0716620127870716
NLA26_RS13345		DUF3885 domain-containing protein	-1.79891941854713	0,02167424	0.368182416670239
NLA26_RS13415		BCCT family transporter	2.53336902564128	0,000246689	0.016891432761617
NLA26_RS13445	sfaB	staphyloferrin A synthetase SfaB	1.62615025158159	0,015419771	0.307238044196202
NLA26_RS13450	sfaC	staphyloferrin A biosynthesis protein SfaC	1.35017825663696	0,043428609	0.51976149267412
NLA26_RS13460		Fe(3+) dicitrate ABC transporter substrate-binding protein	-2.30223283329989	0,000834684	0.0477765879120463
NLA26_RS13465		iron ABC transporter permease	-1.77786399014414	0,009353707	0.224549659034828
NLA26_RS13605		BglG family transcription antiterminator	1.56022653101905	0,019856016	0.346231055404228
NLA26_RS13665		SDR family oxidoreductase	1.94803315681208	0,004204194	0.133369198463475
NLA26_RS13685		thiol-disulfide oxidoreductase DCC family protein	1.69868231934201	0,014217087	0.299608779099668
NLA26_RS13690		DNA starvation/stationary phase protection protein	-2.68337001247475	0,000114076	0.00968906961642474
NLA26_RS13765		UDP-N-acetylglucosamine 1-carboxyvinyltransferase	-1.49702503140308	0,025037723	0.399541368040099
NLA26_RS13775		aldehyde dehydrogenase family protein	1.92400287403149	0,004432776	0.135715108065041
NLA26_RS13880		DUF1146 family protein	1.72965218532618	0,010277198	0.241605911712301
NLA26_RS13885	murA	UDP-N-acetylglucosamine 1-carboxyvinyltransferase	1.62155160754343	0,01551943	0.307238044196202
NLA26_RS13890	fabZ	3-hydroxyacyl-ACP dehydratase FabZ	1.53020046211191	0,022248657	0.371579161326147
NLA26_RS13905	sceD	lytic transglycosylase SceD	-1.94786564944874	0,023091217	0.375303540824621
NLA26_RS13910	tenA	thiaminase II	-2.68393237261938	0,000626438	0.0383584305345717
NLA26_RS13915	thiD	bifunctional hydroxymethylpyrimidine kinase/phosphomethylpyrimidine kinase	-2.66064971885769	0,000250196	0.016891432761617
NLA26_RS13920	thiM	hydroxyethylthiazole kinase	-1.42827242649454	0,044167711	0.521495893842943
NLA26_RS14055		anti-sigma factor antagonist	1.3138116236674	0,048580501	0.553733585865085
NLA26_RS14060	rsbW	anti-sigma B factor RsbW	2.31778083860167	0,000746998	0.0437076636491352
NLA26_RS14065	sigB	RNA polymerase sigma factor SigB	1.37309340945503	0,03916279	0.500177543140077
NLA26_RS14105		hypothetical protein	-3.47435847855496	3,64E-05	0.00416493332519434
NLA26_RS14145	ilvB	biosynthetic-type acetolactate synthase large subunit	2.98838477164332	2,89E-05	0.00365437963903845
NLA26_RS14150	ilvD	dihydroxy-acid dehydratase	3.66737263701704	5,70E-07	0.000125070262286828
NLA26_RS14375	tst	toxic shock syndrome toxin TSST-1	-5.33503531843038	2,70E-11	2.36691929312753e-08
NLA26_RS14420		leukocidin family pore-forming toxin	-2.23387228314261	0,001941889	0.0913034778452748

NLA26_RS14550	DnaD domain protein	2.21155102013028	0,029323086	0.424321446252027

Table S3. Significant transcriptional comparison analysis of all differentially expressed genes in between *S. aureus* MN8*ΔsaeS* with or without PP-HCl

Locus tag	Gene name	Description	Log (Fold Change)	P-Value	False Discovery Rate
NLA26_RS00795		DUF2977 domain-containing protein	2.78063611768704	0,029588432	1
NLA26_RS03830		low specificity L-threonine aldolase	-1.49318649215645	0,034584202	1
NLA26_RS04200	pstA	phosphate ABC transporter permease PstA	1.39047410146421	0,046702422	1
NLA26_RS04205	pstC	phosphate ABC transporter permease subunit PstC	1.9712862157791	0,00578823	1
NLA26_RS04210		phosphate ABC transporter substrate-binding protein PstS	2.44837282757466	0,000665327	0,253679518
NLA26_RS04335	arlR	response regulator transcription factor ArlR	-1.35987390179775	0,041224181	1
NLA26_RS05350		YqgQ family protein	-3.57436010981816	9,03E-07	0,000482246
NLA26_RS05385		metal ABC transporter permease	1.36437070134833	0,041832075	1
NLA26_RS07335		LysR family transcriptional regulator	-2.01540562441738	0,00307557	0,820869601
NLA26_RS08715		YbcC family protein	1.62408062729091	0,015626425	1
NLA26_RS08720		NADH dehydrogenase subunit 5	1.6306280167992	0,016080714	1
NLA26_RS10120		hypothetical protein	1.55222147037021	0,020975148	1
NLA26_RS10820	cna	collagen adhesin Cna	-4.93040097639981	1,83E-10	2,45E-07
NLA26_RS11025	secA2	accessory Sec system translocase SecA2	2.1654265152579	0,001491798	0,44240088
NLA26_RS11215	nrdD	anaerobic ribonucleoside-triphosphate reductase	1.32978526456752	0,045503737	1
NLA26_RS11520		heavy metal translocating P-type ATPase	1.50974210457753	0,023825621	1
NLA26_RS11560	feoB	ferrous iron transport protein B	6.20513636463751	7,86E-14	2,10E-10
NLA26_RS11645		hypothetical protein	2.00770247835495	0,00595953	1
NLA26_RS11690		transposase	1.65941555965039	0,035334578	1
NLA26_RS11890		DUF1433 domain-containing protein	-2.22728831475513	0,001291952	0,431027491
NLA26_RS12220	mdeA	multidrug efflux MFS transporter MdeA	-2.65272687916569	0,00013442	0,059794473
NLA26_RS12500		DUF3021 domain-containing protein	4.21228275356186	3,39E-08	2,26E-05
NLA26_RS12505		LytTR family DNA-binding domain-containing protein	4.95566596092048	3,25E-10	2,89E-07
NLA26_RS12895		urease subunit gamma	2.76911646864187	0,014413706	1
